# Fully Automated Multi-Step Synthesis of Block Copolymers

**DOI:** 10.3390/polym14020292

**Published:** 2022-01-11

**Authors:** Timo Schuett, Julian Kimmig, Stefan Zechel, Ulrich S. Schubert

**Affiliations:** 1 Laboratory of Organic and Macromolecular Chemistry (IOMC), Friedrich Schiller University Jena, Humboldtstr. 10, 07743 Jena, Germany; timo.schuett@uni-jena.de (T.S.); julian.kimmig@uni-jena.de (J.K.); stefan.zechel@uni-jena.de (S.Z.); ulrich.schubert@uni-jena.de (U.S.S.); 2 Jena Center for Soft Matter (JCSM), Friedrich Schiller University Jena, Philosophenweg 7, 07743 Jena, Germany

**Keywords:** automation, automated synthesis, block copolymers, automated dialysis

## Abstract

An automated synthesis protocol is developed for the synthesis of block copolymers in a multi-step approach in a fully automated manner. For this purpose, an automated dialysis setup is combined with robot-based synthesis protocols. Consequently, several block copolymerizations are executed completely automated and compared to the respective manual synthesis. As a result, this study opens up the field of autonomous multi-step reactions without any human interactions.

## 1. Introduction

Block copolymers are macromolecules with at least two different blocks of monomers that are linearly and/or radially connected [1,2]. Due to high variability in composition and properties, block copolymers enable a wide range of potential applications. Amphiphilic block copolymers are applicable as surfactants [3,4] and pigment dispersants [5]. Furthermore, copolymers containing, e.g., poly(ethylene oxide) as hydrophilic block and poly(*L*-amino acid) as hydrophobic block, can aggregate in water to micelles [6]. The core of the micelles can be loaded with a drug for the use as long-circulating drug carriers [7,8].

There are several techniques to synthesize such block copolymers [9]. In order to obtain well-defined products with a small dispersity, living ionic [2,10,11,12] and controlled radical polymerizations [13,14] are the most successful and utilized approaches. Recently, those techniques were utilized in flow chemistry to produce block copolymers rapidly. However, these approaches are limited to monomers that reach a high conversion to avoid purification and isolation protocols [15,16].

Living and controlled polymerizations were also utilized in the past using automated protocols and synthesis robots for the preparation of linear polymers [17]. In particular, controlled living radical polymerizations such as reversible addition–fragmentation chain transfer (RAFT) polymerizations were applied in the past [14,18,19]. Nevertheless, nearly every established polymerization type has also been performed as a high-throughput experimentation (HTE) approach: Step growth [20,21] as well as chain-growth polymerizations [22], living ionic [23] and controlled radical polymerizations [24,25], and even copolymerizations (one-step synthesis) [18,26]. The first approach in this field was published in 1997 by Brocchini and coworkers with the first combinatorial attempt to create a polymer library of 112 biodegradable aromatic polyesters by step-growth polymerization [20]. To accelerate the screening of properties and the optimization of polymerization conditions, HTE was typically performed with an automated synthesizer (synthesis robot) [17]. Furthermore, Haven and coworkers created a quasi-block copolymer library in a one-pot HTE approach utilizing an automated synthesizer. Nevertheless, they still avoided purification steps by assuming a full monomer conversion [27].

To accelerate kinetic investigations, online characterization was realized as well. An automated synthesizer can, in principle, be combined with nearly every characterization device (e.g., nuclear magnetic resonance (NMR)-spectroscopy [28], size exclusion chromatography (SEC) or gas chromatography (GC) [29]). An advantage is the possibility to adjust conditions of the running reaction, such as temperature or time, after analyzing the results of the first samples [30].

In comparison to formulation and characterization, purification is a widely unexplored field. A first approach was studied in 2003 [31,32,33,34]. A copper catalyst of an ATRP reaction was automatically removed via a short column filled with aluminum oxide in a solid phase extraction (SPE) process [32,33,34]. The first automated precipitation was performed in diethyl ether using poly(2-ethyl-2-oxazoline) synthesized via living cationic polymerization [31]. Prior to this study, we developed an automated continuous dialysis process, which is compatible to the automated synthesizer [35]. The system was investigated utilizing tetrahydrofuran (THF) as a solvent, which is commonly used in polymer purification [36,37,38,39] and resulted in a monomer removal up to the detection limit of the NMR-spectrometer in less than 48 h [39].

At the current point of research, automated synthesis [20,25] and characterization [30] are already possible using such robots. However, automated purification techniques directly connected to synthesis robots are rarely investigated [31,32,33,34]. Hence, batchwise multi-step synthesis such as the sequential synthesis of pure block copolymers in high yields is still not possible without having a complete conversion of the monomers.

Consequently, the current investigation focuses on the implementation of a dialysis setup to access the field of automated multi-step reactions (i.e., polymerizations) using a synthesis robot without human intervention. Furthermore, several block copolymerizations are performed manually and automatically to prove the potential of the developed technology.

## 2. Materials and Methods

### 2.1. Materials and Methods

All chemicals were used as received from TCI (Eschborn, Germany), Sigma Aldrich (Darmstadt, Germany), Alfa Aesar (Kandel, Germany), VWR (Darmstadt, Germany), Thermo Fisher Scientific and Acros Organics (Schwerte, Germany) if not otherwise stated. Dimethylformamide (DMF) (99.8%) was dried over molecular sieve under nitrogen atmosphere. The used liquid monomers, butyl acrylate (≥99%) and styrene (≥99%), were destabilized using a short AlOx column (neutral AlOx, obtained from Molekula (Munich, Germany). The dialysis tubings were purchased from Spectrum Labs (Ravensburg, Germany) (Spectra/Por^TM^, pre-wetted tubing, 3.5 kDa) and rinsed with THF (99%) before use.

Nuclear magnetic resonance spectra (^1^H, DOSY) were recorded on a Bruker AV 250 (250 MHz), Bruker AV 400 (400 MHz) and a Bruker AV 500 (500 MHz) spectrometer at 298 K. The chemical shifts are given in parts per million (ppm on *δ* scale) related to deuterated solvent.

Size exclusion chromatography measurements were performed on the following setup: Shimadzu with CBM-20A (system controller), DGU-14A (degasser), LC-20AD (pump), SIL-20AHT (auto sampler), CTO-10AC vp (oven), SPD-20A (UV detector), RID-10A (RI detector), and PSS SDV guard/1000 Å/1,000,000 Å (5 μm particle size) chloroform/isopropanol/triethylamine [94/2/4] with 1 mL min^−1^ at 40 °C, poly(methyl methacrylate) or polystyrene (standard).

The automated polymerizations and purifications were performed using a Chemspeed SLT-Accelerator automated parallel synthesizer (Basel, Switzerland) equipped with an overhead robot arm that can pick up several tools such as a four-needle head (4-NH) for automated liquid handling of solvents and a glass reactor block, as well as an individual heater. The synthesizer is equipped with a glass reactor block consisting of four reaction vessels (60 mL) with thermal jackets and reflux condensers connected in series through the reaction block and connected to a heating/cooling system (Unistat Tango, heating range: −40 to 145 °C). The synthesis robot was programmed utilizing the AutoSuite software (Version 2.2) by Chemspeed (AutoSuite Editor, AutoSuite Executer, and AutoSuite Driver Manager).

### 2.2. Synthesis of the Polymers

The synthesis of all polymers (**P1**–**P4**) was adapted from a literature procedure [40] and is reported in more details in the Supporting Information. An overview of all synthesized polymers and copolymers is shown in Table 1. An exemplary synthesis protocol for one manual (**P3**) and one automated approach (**P4**) is described in the following.

#### 2.2.1. Manual Synthesis of **P3c**

The pentablock copolymer **P3c** was synthesized via homopolymerization of styrene (resulting in **P3a**). Subsequently, **P3a** was converted to **P3b** (triblock) and finally to **P3c** (pentablockcopolymer).

Styrene (10.0 g, 96.0 mmol), azobisisobutyronitrile (AIBN) (26.3 mg, 0.16 mmol), and *S,S*-dibenzyl trithiocarbonate (DBTTC) (185.9 mg, 0.64 mmol) were dissolved in dimethylformamide (DMF) in a 50 mL round bottom flask ([M]:[CTA]:[I] ratio of 150:1:0.25) (M = monomer; CTA = chain-transfer agent; I = initiator). After closing the flask with a septum, the solution was degassed for 30 min with nitrogen. The solution polymerization was carried out in a pre-heated oil-bath at 70 °C for 17 h. Polystyrene (PS, **P3a**) (3.0 g, 30%) was obtained after dialysis in THF (5 × 350 mL, solvent exchange after 12 h) and after drying in vacuo.

**^1^H NMR** (250 MHz, CDCl_3_,): 0.87–2.55 (3H), 6.32–7.55 (5H) ppm.

**SEC** (chloroform/isopropanol/triethylamine [94/2/4], PS standard): M_n_ = 5900 g mol^−^^1^, M_w_ = 7500 g mol^−^^1^, Ð = 1.28.

Subsequently, **P3a** (2.87 g, 0.49 mmol), AIBN (20.0 mg, 0.12 mmol) and butyl acrylate (BA) (9.4 g, 72.9 mmol) were dissolved in DMF in a 25 mL round bottom flask ([M]:[CTA]:[I] ratio of 150:1:0.25). After closing the flask with a septum, the solution was degassed for 30 min with nitrogen. The solution polymerization was carried out in a pre-heated oil-bath at 70 °C for 17 h. PBA-*b*-PS-*b*-PBA (**P3b**) (7.33 g, 60%) was obtained after dialysis in THF (5 × 350 mL, solvent exchange after 12 h) and after drying in vacuo.

**^1^H NMR** (250 MHz, CDCl_3_,): 0.74–1.08 (3H), 1.09–2.57 (12H), 3.36–4.10 (2H), 6.30–7.58 (9H) ppm.

**SEC** (chloroform/isopropanol/triethylamine [94/2/4], PS standard): M_n_ = 17,800 g mol^−^^1^, M_w_ = 21,500 g mol^−1^, Ð = 1.20.

Afterwards, **P3b** (7.33 g, 0.41 mmol), AIBN (16.6 mg, 0.10 mmol), and styrene (6.33 g, 60.78 mmol) were dissolved in DMF in a 25 mL round bottom flask ([M]:[CTA]:[I] ratio of 150:1:0.25). After closing the flask with a septum, the solution was degassed for 30 min with nitrogen. The solution polymerization was carried out in a pre-heated oil-bath at 70 °C for 17 h. **P3c** (6.89 g, 50%) was obtained after dialysis in THF (5 × 350 mL, solvent exchange after 12 h) and after drying in vacuo.

**^1^H NMR** (250 MHz, CDCl_3_,): 0.78–1.05 (3H), 1.10–2.48 (16H), 3.35–4.11 (2H), 6.29–7.49 (14H) ppm.

**SEC** (chloroform/isopropanol/triethylamine [94/2/4], PS standard): M_n_ = 20,800 g mol^−^^1^, M_w_ = 26,000 g mol^−1^, Ð = 1.25.

#### 2.2.2. Automated Synthesis of **P4c**

The pentablock copolymer **P4c** was synthesized via homopolymerization of styrene (resulting in **P4a**). Afterwards, **P4a** was converted to **P4b** (triblock) and finally to **P4c** (pentablock copolymer).

Styrene (10 g, 96.0 mmol), AIBN (26.3 g, 0.16 mmol) and DBTTC (185.9 g, 0.64 mmol) were dissolved via the 4-NH in DMF in a 60 mL glass reactor ([M]:[CTA]:[I] ratio of 150:1:0.25). The solution was degassed for 30 min with nitrogen and the polymerization was carried out by heating the oil-jacket to 70 °C for 17 h. Polystyrene (**P4a**) (3.0 g, 30%) was obtained after dialysis in THF (35 mL/h, 32 h) and after drying in vacuo.

**^1^H NMR** (250 MHz, CDCl_3_,): 1.02–2.88 (3H), 6.54–7.99 (5H) ppm.

**SEC** (chloroform/isopropanol/triethylamine [94/2/4], PS standard): M_n_ = 5200 g mol^−^^1^, M_w_ = 6700 g mol^−^^1^, Ð = 1.28.

Then, **P4a** (3.0 g, 0.6 mmol) dissolved in THF was transferred automatically from the dialysis tubing to a second reactor and the solvent was evaporated in vacuo. AIBN (22.8 mg, 0.14 mmol) and butyl acrylate (10.7 g, 83.5 mmol) were dissolved in DMF in a 60 mL glass reactor ([M]:[CTA]:[I] ratio of 150:1:0.25). The solution was degassed for 30 min with nitrogen and the polymerization was carried out by heating the oil-jacket to 70 °C for 17 h. **P4b** (7.0 g, 51%) was obtained after dialysis in THF (35 mL/h, 32 h) and after drying in vacuo.

**^1^H NMR** (250 MHz, CDCl_3_,): 0.89–1.14 (3H), 1.22–2.21 (7H), 4.41–4.20 (1H), 6.41–7.62 (19H) ppm.

**SEC** (chloroform/isopropanol/triethylamine [94/2/4], PS standard): M_n_ = 13,700 g mol^−^^1^, M_w_ = 17,100 g mol^−^^1^, Ð = 1.25.

Afterwards, **P4b** (7.0 g, 0.51 mmol) dissolved in THF was transferred automatically from the dialysis tubing to a second reactor and the solvent was evaporated in vacuo. AIBN (21.8 mg, 0.13 mmol) and styrene (8.3 g, 79.7 mmol) were dissolved in DMF in a 60 mL glass reactor ([M]:[CTA]:[I] ratio of 150:1:0.25). The solution was degassed for 30 min with nitrogen and the polymerization was carried out by heating the oil-jacket to 70 °C for 17 h. **P4c** (11.4 g, 76%) was obtained after dialysis in THF (35 mL/h, 32 h) and after drying in vacuo.

**^1^H NMR** (250 MHz, CDCl_3_,): 0.64–0.95 (3H), 0.98–2.37 (14H), 3.24–4.03 (2H), 6.19–7.41 (12H).

**SEC** (chloroform/isopropanol/triethylamine [94/2/4], PS standard): M_n_ = 22,200 g mol^−^^1^, M_w_ = 26,800 g mol^−^^1^, Ð = 1.21.

#### 2.2.3. General Procedure of Manual Dialysis

The polymer was dissolved in THF (100 mg/mL), poured into a dialysis tubing (3.5 kDa, d = 34 mm) and stored in a beaker filled with 350 mL THF. The surrounding solvent was exchanged every 12 h and a new 400 mL of THF was utilized for this purpose.

#### 2.2.4. Automated Dialysis

The procedure was performed according to literature [35]. The polymer was dissolved in THF (100 mg/mL) and poured into dialysis tubing (3.5 kDa, d = 34 mm) using the 4-NH of the robot. The tubing was fixed to the cap of the dialysis system. The surrounding solvent (250 mL THF) was continuously exchanged by a pump with a volume flow of 35 mL/h. After 48 h, the purified polymeric solution was extracted and the dialysis tubing was rinsed with THF (10 × 10 mL).

#### 2.2.5. Reusability Test of Dialysis Tubing

An automated dialysis setup was prepared as mentioned above. Fifteen milliliters of the raw reaction solution of 1 g PMMA (**P5**) in THF was filled into the tubing and a continuous solvent flow of 35 mL/min was applied for 24 h. Afterwards, the purified polymeric solution was extracted utilizing a pump and rinsed four times with 10 mL THF for each cycle using the 4-NH of the synthesis robot. Each fraction was collected and analyzed via SEC. Subsequently, 10 mL of a solution of 1 g PS (**P6**) in THF was filled into the rinsed dialysis tubing. After 24 h, the polymer was extracted, the dialysis tubing was rinsed and 1.5 mL of each rinsing fraction was analyzed via SEC.

## 3. Results and Discussion

As a first experiment, the copolymerization of the triblock copolymer (**P1b**, PS-*b*-PBA-*b*-PS) and the pentablock copolymer (**P3b**, PS-*b*-PBA-*b*-PS*-b-*PBA-*b*-PS) was performed manually in order to generate reference data for the automated setup. The polymerization was inspired by a literature example of the RAFT-polymerization of multi-block copolymers [40]. Thus, styrene was polymerized first (**P1a**, **P2a**, **P3a**, **P4a**), followed by a synthesis of the triblock copolymer using butyl acrylate as second monomer (**P1b**, **P2b**, **P3b**, **P4b**) and finally the polymerization of a pentablock copolymer using styrene again as monomer (**P3c**, **P4c**). The reaction was performed using standard round bottom flasks equipped with septa and a typical dialysis setup using beakers and THF as solvent. The resulting molar masses, dispersities Ð and yields are summarized in Table 1.

**Figure 1:**
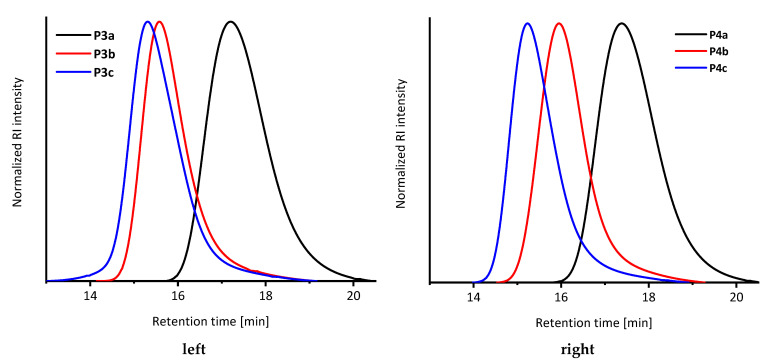
SEC-Elugram of the manually (**P3**, **left**) and automated (**P4**, **right**) synthesized block copolymer. Black: First reaction step (PS, **P3a**/**P4a**). Red: Second reaction step (PS-*b*-PBA-*b*-PS, **P3b**/**P4b**). Blue: Third reaction step (PS-*b*-PBA-*b*-PS-*b*-PBA-*b*-PS, **P3c**/**P4c**). Standard: Poly(methyl methacrylate) or polystyrene; solvent: chloroform/isopropanol/triethylamine [94/2/4]).

As expected, the molar mass increased within every synthesis step (Figure 1, left; **P3a**: M_n_ = 5900 g mol^−^^1^, **P3b**: M_n_ = 17,800 g mol^−^^1^, **P3c**: M_n_ = 20,800 g mol^−^^1^). All dispersities are very low, indicating a good control over the consecutive polymerizations.

**Figure 2:**
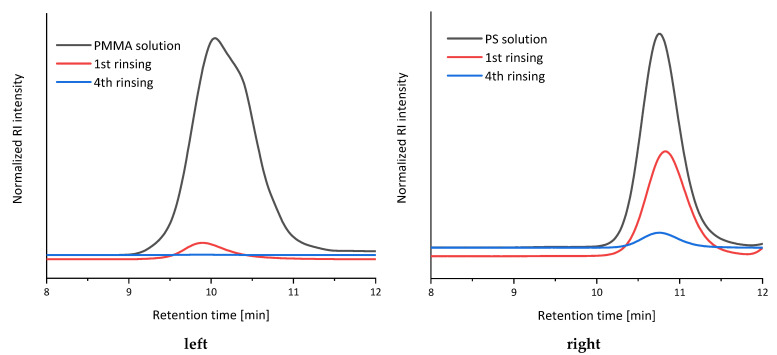
SEC-Elugram of dialysis tubing reusability tests. (**left**) PMMA (**P5**) solution. (**right**) PS (**P6**) solution. Black: Raw polymeric solution. Red: 1st rinsing fraction. Blue: 4th rinsing fraction.

Secondly, due to limited space in the synthesis robot, the reusability of a dialysis tubing was tested. Therefore, two typical automated dialysis approaches were performed each for 24 h in the same dialysis tubing utilizing the prior developed setup [35] and two different polymers synthesized for this approach (**P5**–**P6**). After each purification, the dialysis tubing was rinsed for four cycles by inserting and extracting THF. Subsequently, every rinsing solvent fraction was analyzed via SEC to investigate the residual polymer. The resulting elugrams are shown in Figure 2. The dialysis tubing filled with PMMA (**P5**) dissolved in THF revealed no residual polymer after the fourth rinsing step (Figure 2, left). Compared to this, there was a small polystyrene (**P6**) signal after the fourth rinsing step in the respective experiment (Figure 2, right). As a consequence, the dialysis tubing was rinsed in 10 cycles after each reaction step of the copolymerizations, to ensure that there are no contaminations with polymer of the prior synthesis. Additionally, a prior long-term experiment revealed the usability of dialysis tubing for a long period of time [35]. Thus, those tubings can be applied for more than 28 d.

Afterwards, the automated setup was installed to ensure a completely autonomous working system. For this purpose, the stock solutions (AIBN and DBTTC, each in DMF), the respective monomer (styrene or butyl acrylate) as well as the solvent (DMF) were fixed in the stock solution rack inside the synthesis robot. Furthermore, the automated dialysis setup was installed.

For this purpose, the dialysis tubing was equipped with a Teflon hemisphere at the bottom to increase the volume of the tubing filled with air, which, in consequence, prevents it from overfilling and piercing of the tubing with the needle of the robot.

Additionally, a rinsing solvent (THF) for the dialysis tubing was placed inside the robot to enable purification of the dialysis tubing between two purification steps.

After the automated synthesis and purification of **P2a**, a specific volume was taken for analysis and calculation of the yield. Based on the results, the second step (**P2b**) was calculated, programmed, and performed automatically. To enable no human interactions during the synthesis of **P4**, a theoretical yield of the PS-block of 30% and of the BA block of 40% was presumed for the addition of the substrates of the respective next step. The assumed yields are based on the findings of the manual reactions.

**Scheme 1:**
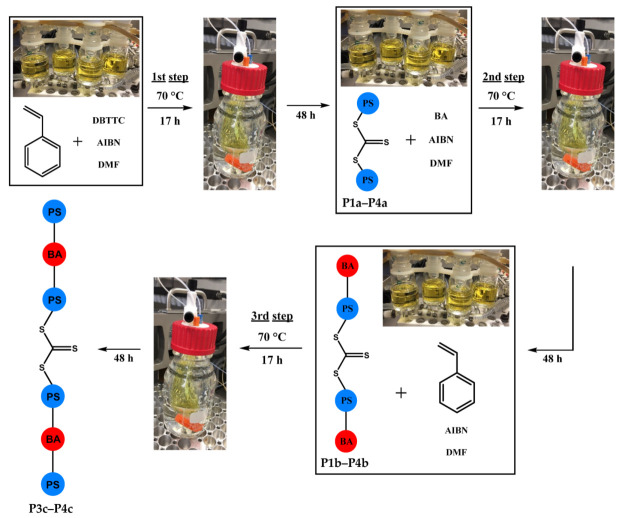
Schematic representation of the automated synthesis of PS-*b*-PBA-*b*-PS-*b*-PBA-*b*-PS, utilizing the developed automated dialysis system.

According to Scheme 1, the triblock copolymer (PS-*b*-PBA-*b*-PS) **P2b** and the pentablock copolymer (PS-*b*-PBA-b-PS-*b*-PBA-*b*-PS) **P4c** were synthesized in a fully automated manner. After each purification, a specific volume was extracted out of the process for characterization and calculation of the yield.

All polymers were characterized using SEC and NMR spectroscopy, which are depicted in the SI. The SEC traces indicates a successful synthesis of the block copolymers due to a shift to lower elution volumes (Table 1, Figure 1).

Furthermore, no significant tailing could be observed indicating the formation of the block copolymers. Within the NMR spectra, the signals of the corresponding moieties could be found (i.e., styrene and butyl acrylate) The ratio between these signals changes during the formation of block copolymer structures. Furthermore, it could be shown that for the final products, no residual monomer is observable beside polymer **P4c**, for which slightly monomer signals could be observed.

**Figure 3:**
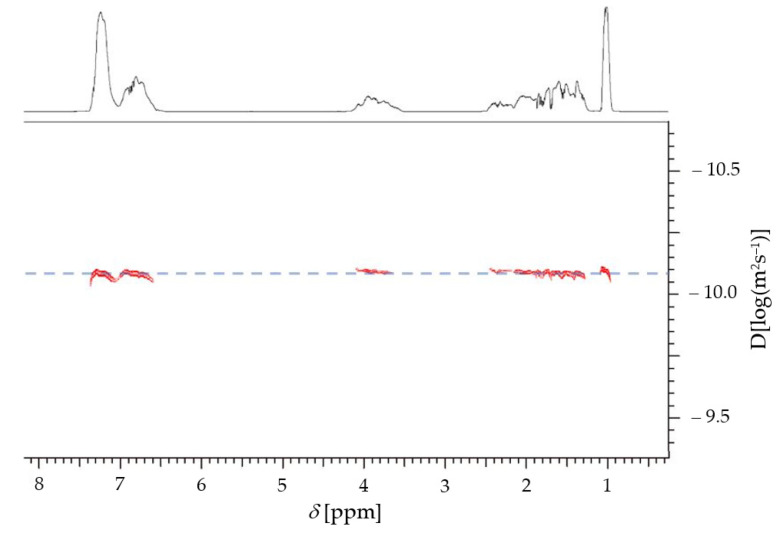
DOSY-NMR spectrum of PS-*b*-PBA-*b*-PS-*b*-PBA-*b*-PS (**P4c**) in CDCl_3_ (500 MHz).

Furthermore, the dispersity of all polymerizations is low (below 1.3), indicating a high control of the polymerizations. To ensure there is only one polymer species, ^1^H-DOSY measurements were performed with the block copolymer samples (**P1b**, **P2b**, **P3b**, **P3c**, and **P4c**, respectively). An example spectrum is shown in Figure 3; all other spectra can be found in the Supplementary Information. As it can be seen, there is only one species in the solution and, thus, the synthesis of the block copolymer was successful for both approaches. All NMR spectra and SEC-curves can be found in the Supplementary Information.

In comparison, both the manual as well as the automated approach feature a similar yield, purity, dispersity, and molar mass. However, the amount of work is significantly decreased in the automated procedure. After preparing the robot, by writing the program, inserting the substrates, and preparing the dialysis setup, no further interaction was required to achieve a purified pentablock copolymer.

Furthermore, the automated approach led faster to the destined product due to a more efficient dialysis and the robot working every day and night (total amount of time for the pentablock copolymer synthesis: Automated: 9 d, manual: 10 d). The total amount of time for both approaches is such high due to the purification via dialysis. A further improvement in the future can be the utilization of other techniques such as precipitation.

However, the amount of solvent required for the manual approach is lower compared to the automatic one (automated: 7.5 L, manual: 5.25 L). Nevertheless, the automated purification of the dialysis tubings resulted in a significant advantage since only one tubing is required per block copolymer synthesis.

## 4. Conclusions

In summary, a new, fully automated protocol was developed enabling the sufficient synthesis of multi-block copolymers via the reversible-addition-fragmentation chain-transfer (RAFT)-polymerization.

For this purpose, a dialysis system was combined with a synthesis robot. Consequently, the synthesis and the purification of each block could be performed without any human intervention.

In comparison to the manual approach, automated block copolymerization decreases the amount of human work significantly, is faster and leads to comparable results regarding purity, yield, molar mass, and dispersity. However, the yield and purity of every step have to be estimated before the synthesis to start the automated program and the amount of solvent needed for automated dialysis is slightly higher than for manual dialysis (automated: 7.5 L, manual: 5.25 L). Therefore, further optimizations will be performed in order to improve the system. Furthermore, investigations on the dialysis setup regarding type of solvent, polymer and monomer type as well as size and the cut-off of the dialysis tubing are currently in progress.

Overall, the new setup integrating an automated purification technique into a continuous workflow enables automated multi-step synthesis in a fully automated manner.

## Figures and Tables

**Table 1:** Summary of the molar masses of the synthesized polymers **P1**–**P4** (the molar masses were determined using SEC; standard: Polystyrene; solvent: Chloroform/isopropanol/triethylamine [94/2/4]).

Number	Polymer	Synthesis Approach	M_n_ (g mol^−1^)	M_w_ (g mol^−1^)	Đ	Yield	
						[g]	[%]
**P1a**	PS	manual	3800	4900	1.29	1.00	20
**P1b**	PS-*b*-PBA-*b*-PS	manual	8100	10,500	1.30	0.31	17
**P2a**	PS	automated	4100	5100	1.25	0.78	16
**P2b**	PS-*b*-PBA-*b*-PS	automated	12,700	18,700	1.48	0.22	8
**P3a**	PS	manual	5900	7500	1.28	3.00	30
**P3b**	PS-*b*-PBA-*b*-PS	manual	17,800	21,500	1.20	7.33	60
**P3c**	PS-*b*-PBA-*b*-PS-*b*-PBA-*b*-PS	manual	20,800	26,000	1.25	6.89	50
**P4a**	PS	automated	5200	6700	1.28	3.00	30
**P4b**	PS-*b*-PBA-*b*-PS	automated	13,700	17,100	1.25	7.00	51
**P4c**	PS-*b*-PBA-*b*-PS-*b*-PBA-*b*-PS	automated	22,200	26,800	1.21	11.40	76

## Data Availability

The data presented in this study are available on request from the corresponding author.

## Supplementary Materials

The following supporting information can be downloaded at https://www.mdpi.com/article/10.3390/polym14020292/s1, Table S1: Reaction details for the RAFT-polymerizations. Table S2: Summary of the utilized amounts and volumes for the RAFT-polymerizations of MMA and PS. Table S3: Summary of the molar masses of the polymers **P5**–**P6** (molar mass was determined using SEC; standard PMMA for **P5** and polystyrene for **P6**; solvent: Chloroform/isopropanol/triethylamine [94/2/4]). Figure S1: ^1^H NMR spectrum of polymer **P1b** (250 MHz, CDCl_3_). Figure S2: ^1^H NMR spectrum of polymer **P2b** (250 MHz, CDCl_3_). Figure S3: ^1^H NMR spectrum of polymer **P3c** (500 MHz, CDCl_3_). Figure S4: ^1^H NMR spectrum of polymer **P4c** (500 MHz, CDCl_3_). Figure S5: ^1^H NMR spectrum of polymer **P6** (300 MHz, CDCl_3_). Figure S6: DOSY-NMR spectrum of PS-*b*-PBA-*b*-PS (**P1b**) in CDCl_3_ (400 MHz). Figure S7: DOSY-NMR spectrum of PS-*b*-PBA-*b*-PS (**P2b**) in CDCl_3_ (400 MHz). Figure S8: DOSY-NMR spectrum of PS-*b*-PBA-*b*-PS (**P3b**) in CDCl_3_ (400 MHz). Figure S9: DOSY-NMR spectrum of PS-*b*-PBA-*b*-PS-*b*-PBA-*b*-PS (**P3c**) in CDCl_3_ (500 MHz). Figure S10: DOSY-NMR spectrum of PS-*b*-PBA-*b*-PS-*b*-PBA-*b*-PS (**P4c**) in CDCl_3_ (500 MHz). Figure S12: SEC-curve of the automatically synthesized block copolymer **P2**. Black: First reaction step (PS, **P2a**). Red: Second reaction step (PS-*b*-PBA-*b*-PS, **P2b**), (chloroform/isopropanol/triethylamine [94/2/4], PS standard). Figure S13: SEC-curve of the manually synthesized block copolymer **P3**. Black: First reaction step (PS, **P3a**). Red: Second reaction step (PS-*b*-PBA-*b*-PS, **P3b**). Blue: Third reaction step (PS-*b*-PBA-*b*-PS-*b*-PBA-*b*-PS, **P3c**), (chloroform/isopropanol/triethylamine [94/2/4], PS standard). Figure S14: SEC-curve of the automatically synthesized block copolymer **P4**. Black: First reaction step (PS, **P4a**). Red: Second reaction step (PS-*b*-PBA-*b*-PS, **P4b**). Blue: Third reaction step (PS-*b*-PBA-*b*-PS-*b*-PBA-*b*-PS, **P4c**), (chloroform/isopropanol/triethylamine [94/2/4], PS standard). Figure S15: SEC-curves of the synthesized polymers **P5** and **P6**. Red: PMMA, **P5**. Black: PS, **P6**) (chloroform/isopropanol/triethylamine [94/2/4], PMMA or PS standard).
