# Supplementary material for: Fully Automated Multi-Step Synthesis of Block Copolymers
Source: Polymers (Basel). 2022 Jan 11;14(2):292. doi: 10.3390/polym14020292 (PMC8780857; doi:10.3390/polym14020292)
Supplement: Supplementary file 1 [file polymers-14-00292-s001.zip › polymers-1529708-supplementary.pdf]

polymers

Supporting Information

Fully Automated Multi-Step Synthesis of Block Copolymers

Timo Schuett [1,2], Julian Kimmig [1,2], Stefan Zechel [1,2] and Ulrich S. Schubert [1,2,*]

[1] Laboratory of Organic and Macromolecular Chemistry (IOMC), Friedrich Schiller University Jena, Humboldtstr. 10, 07743 Jena, Germany; timo.schuett@uni-jena.de (T.S.); julian.kimmig@uni-jena.de (J.K.); stefan.zechel@uni-jena.de (S.Z.)
[2] Jena Center for Soft Matter (JCSM), Friedrich Schiller University Jena, Philosophenweg 7, 07743 Jena, Germany
* Correspondence: ulrich.schubert@uni-jena.de

S1. Synthesis

All polymers were synthesized as described in the manuscript. The reaction details for every reaction can be found in Table S1.

Table S1: Reaction details for the RAFT-polymerizations.

Nr.	Polymer	Monomer	RAFT-agent	RAFT-agent	AIBN	Monomer	DMF [mL]
**P1a**	PS	Styrene	DBTTC	93 mg<br>0.32 mmol	13 mg<br>0.08 mmol	5000 mg<br>48 mmol	24
**P1b**	PS-*b*-BA-*b*-PS	BA	**P1a**	250 mg<br>0.08 mmol	3.4 mg<br>0.02 mmol	1600 mg<br>12 mmol	6
**P2a**	PS	Styrene	DBTTC	93 mg<br>0.32 mmol	13 mg<br>0.08 mmol	5000 mg<br>48 mmol	24
**P2b**	PS-*b*-BA-*b*-PS	BA	**P2a**	387 mg<br>0.12 mmol	5 mg<br>0.03 mmol	2400 mg<br>19 mmol	9
**P3a**	Styrene	Styrene	DBTTC	186 mg<br>0.64 mmol	26 mg<br>0.16 mmol	10,000 mg<br>96 mmol	19
**P3b**	PS-*b*-BA-*b*-PS	BA	**P3a**	2,86 mg<br>0.49 mmol	20 mg<br>0.12 mmol	9350 mg<br>73 mmol	24
**P3c**	PS-*b*-BA-*b*-PS-*b*-BA-*b*-PS	Styrene	**P3b**	7333 mg<br>0.41 mmol	17 mg<br>0.10 mmol	6330 mg<br>61 mmol	24
**P4a**	Styrene	Styrene	DBTTC	186 mg<br>0.64 mmol	26 mg<br>0.16 mmol	10,000 mg<br>96 mmol	19
**P4b**	PS-*b*-BA-*b*-PS	BA	**P4a**	3005 mg<br>0.56 mmol	23 mg<br>0.14 mmol	10,700 mg<br>83 mmol	24
**P4c**	PS-*b*-BA-*b*-PS-*b*-BA-*b*-PS	Styrene	**P4b**	7016 mg<br>0.51 mmol	21 mg<br>0.13 mmol	8000 mg<br>77 mmol	31

P1a: ^{1}H NMR (250 MHz, $CDCl_3$, δ): 0.84–2.52 (3H), 6.28–7.53 (5H) ppm.
P1b: ^{1}H NMR (250 MHz, $CDCl_3$, δ): 0.79-1.06 (3H), 1.13–2.55 (10H), 3.70–4.32 (2H), 6.30–7.43 (5H) ppm.
P2a: ^{1}H NMR (250 MHz, $CDCl_3$, δ): 0.84–2.51 (3H), 6.27–7.42 (5H) ppm.
P2b: ^{1}H NMR (250 MHz, $CDCl_3$, δ): 0.80-1.07 (3H), 1.19–2.53 (8H), 3.62–4.21 (3H), 6.30–7.37 (1H) ppm.
P3a: ^{1}H NMR (250 MHz, $CDCl_3$, δ): 0.87–2.55 (3H), 6.32–7.55 (5H) ppm.
P3b: ^{1}H NMR (250 MHz, $CDCl_3$, δ): 0.74-1.08 (3H), 1.09–2.57 (12H), 3.36–4.10 (2H), 6.30–7.58 (9H) ppm.
P3c: ^{1}H NMR (500 MHz, $CDCl_3$, δ): 0.78-1.05 (3H), 1.10–2.48 (16H), 3.35–4.11 (2H), 6.29–7.49 (14H) ppm.
P4a: ^{1}H NMR (250 MHz, $CDCl_3$, δ): 1.02–2.88 (3H), 6.54–7.99 (5H) ppm.
P4b: ^{1}H NMR (400 MHz, $CDCl_3$, δ): 0.89-1.14 (3H), 1.22–2.21 (7H), 4.41–4.20 (1H), 6.41–7.62 (19H) ppm.
P4c: ^{1}H NMR (500 MHz, $CDCl_3$, δ): 0.64-0.95 (3H), 0.98–2.37 (14H), 3.24–.03 (2H), 6.19–7.41 (12H) ppm.

S2. RAFT-Polymerization of methyl methacrylate (P5 – P6)

Solutions of the initiator (azobisisobutyronitrile, AIBN), chain-transfer-agent (2-cyano-2-propylbenzodithioat) and methyl methacrylate (MMA, **P5**) or styrene (**P6**) in DMF were prepared with a [M]:[CTA]:[I] ratio of 150:1:0.25 in a round bottom flask. After closing the reaction vessel with a septum, the reaction mixture was degassed by flushing with nitrogen for 30 minutes. The polymerizations were carried out in a pre-heated oil-bath at 70 °C for 17 h. All amounts and volumes are listed in Table S2.

P5 was utilized for dialysis experiments without further purification. **P6** was precipitated in diethyl ether. The obtained molar masses are summarized in Table S3.

Table S2. Summary of the utilized amounts and volumes for the RAFT-polymerizations of MMA and PS.

Polymer	m(monomer) [g]	V(DMF) [mL]	m(CTA) [mg]	m(AIBN) [mg]
**P5**	15	75	221.07	41.00
**P6**	15	78	88.55	10.00

P6: ^{1}H NMR (300 MHz, $CDCl_3$, δ): 1.35 (m, 2H), 1.76 (m, 1H), 6.24–6.68 (m, 2H), 6.80–7.26 (m, 3H) ppm.

Table S3: Summary of the molar masses of the polymers **P5 – P6** (molar mass was determined using SEC; standard PMMA for **P5** and polystyrene for **P6**; solvent: Chloroform/isopropanol/triethylamine [94/2/4]).

Polymer	M_n (g/mol)	M_w (g/mol)	Đ
**P5**	12,600	14,300	1.13
**P6**	3100	3500	1.13

S2.1. NMR spectroscopy

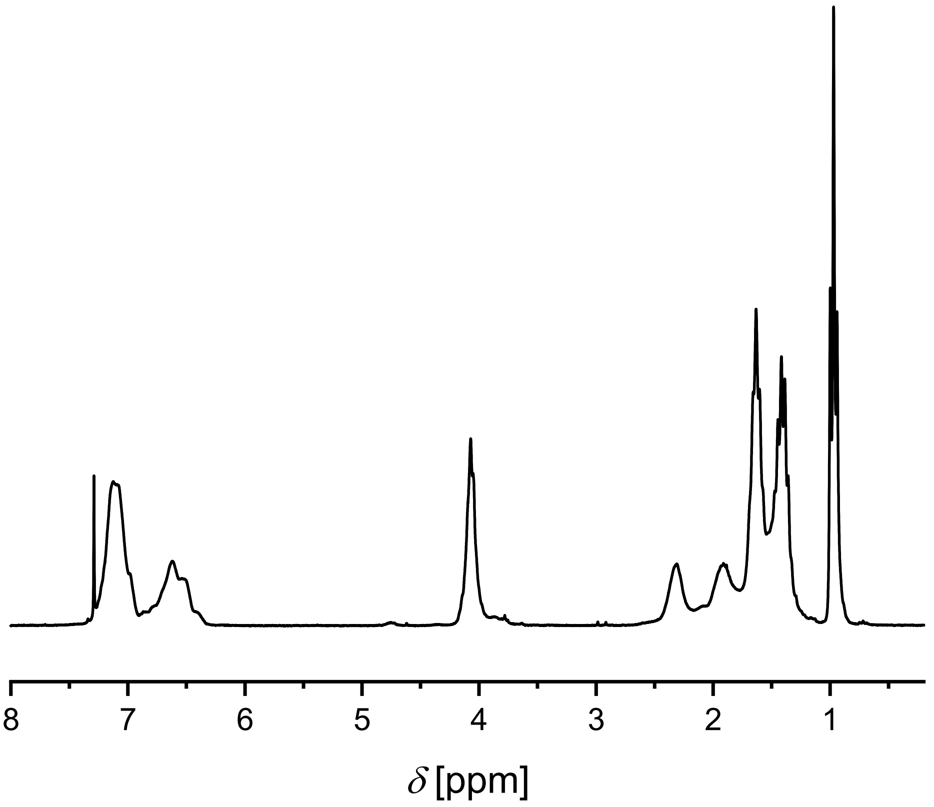

Figure S1: ^{1}H NMR spectrum of polymer **P1b** (250 MHz, $CDCl_3$).

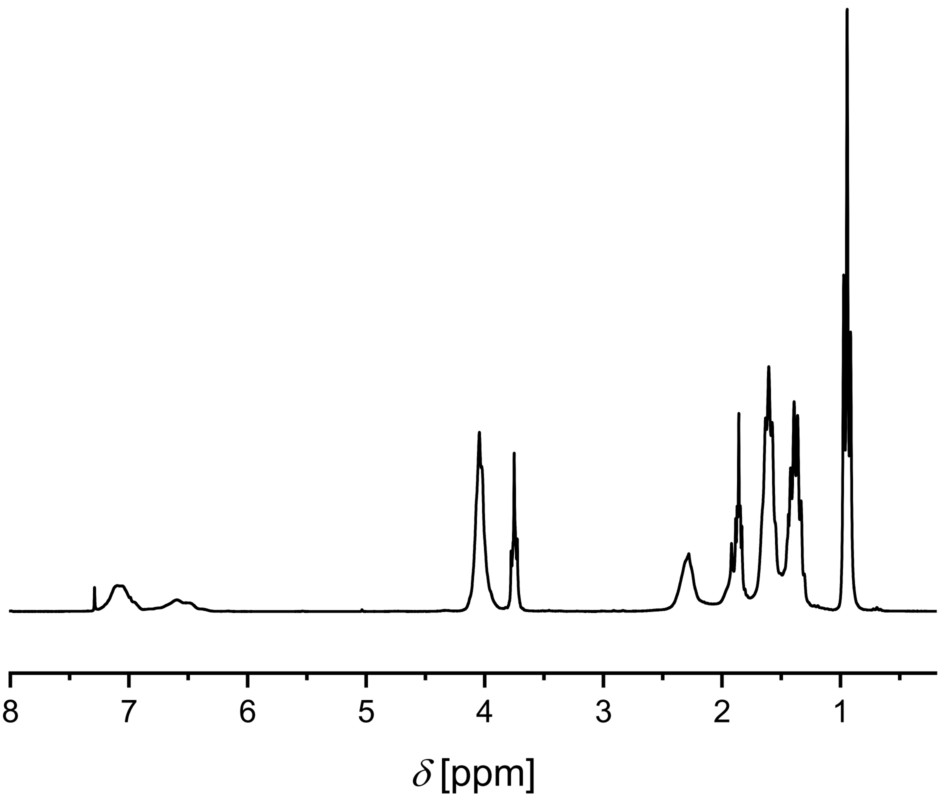

Figure S2: ¹H NMR spectrum of polymer **P2b** (250 MHz, $CDCl_3$).

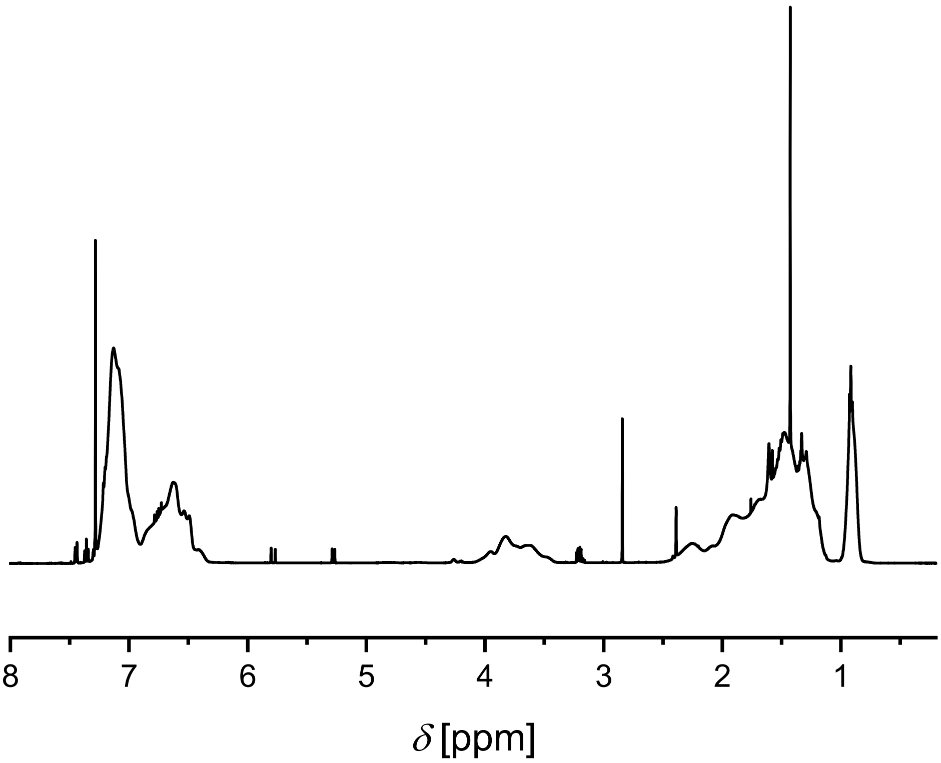

Figure S3: ¹H NMR spectrum of polymer **P3c** (500 MHz, $CDCl_3$).

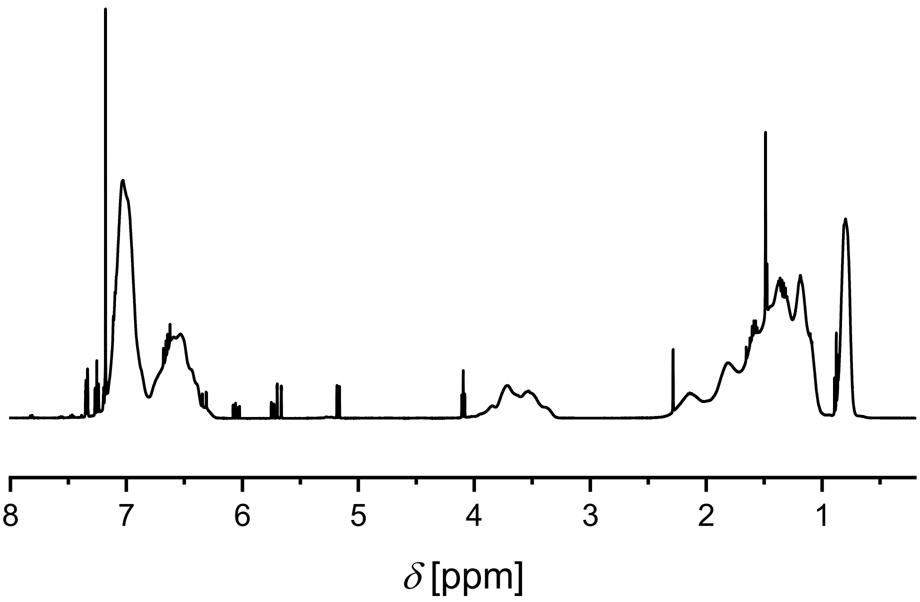

Figure S4: ^{1}H NMR spectrum of polymer **P4c** (500 MHz, $CDCl_3$).

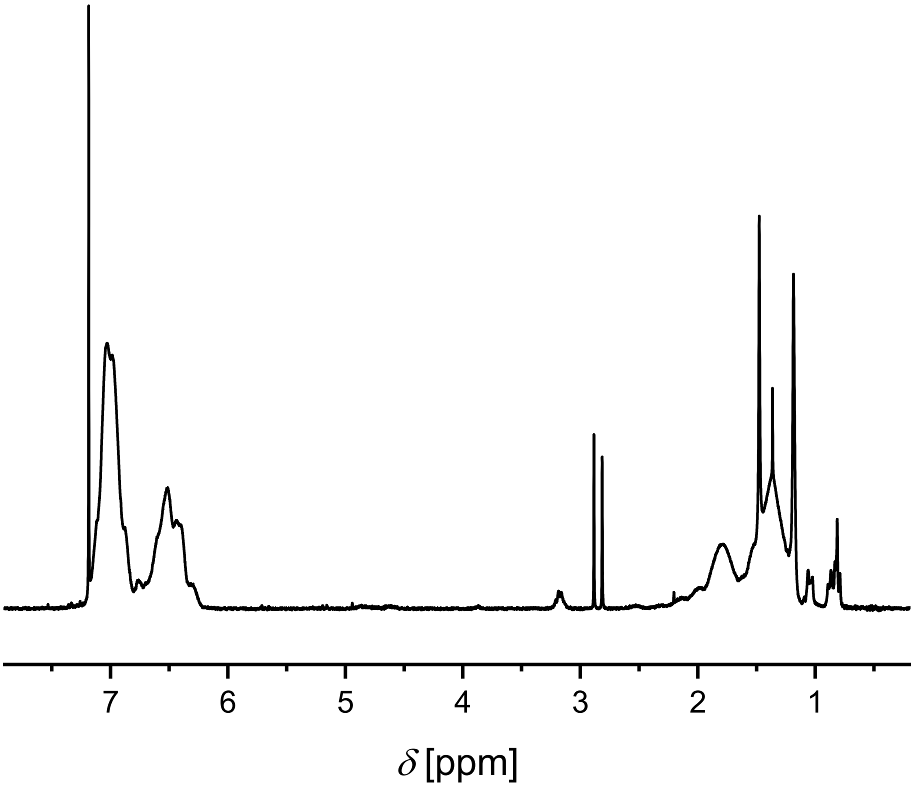

Figure S5: ^{1}H NMR spectrum of polymer **P6** (300 MHz, $CDCl_3$).

S2.2. DOSY-NMR experiments

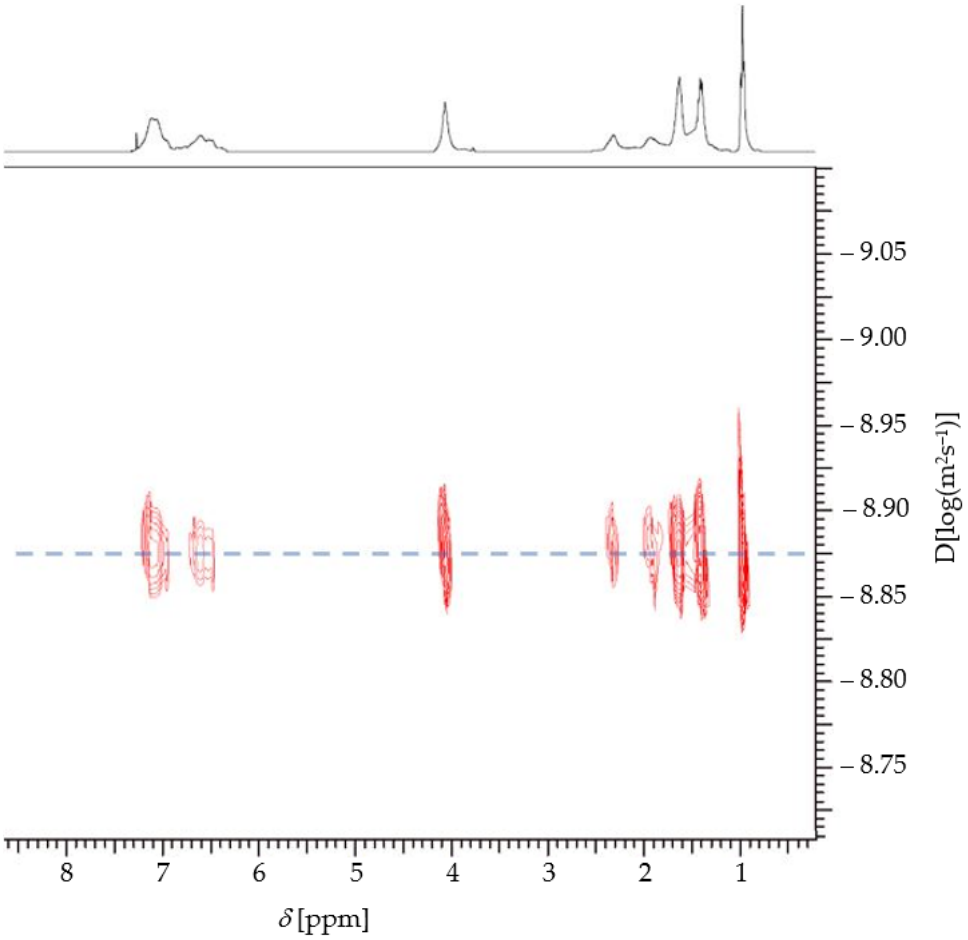

Figure S6: DOSY-NMR spectrum of PS-*b*-PBA-*b*-PS (**P1b**) in $CDCl_3$ (400 MHz).

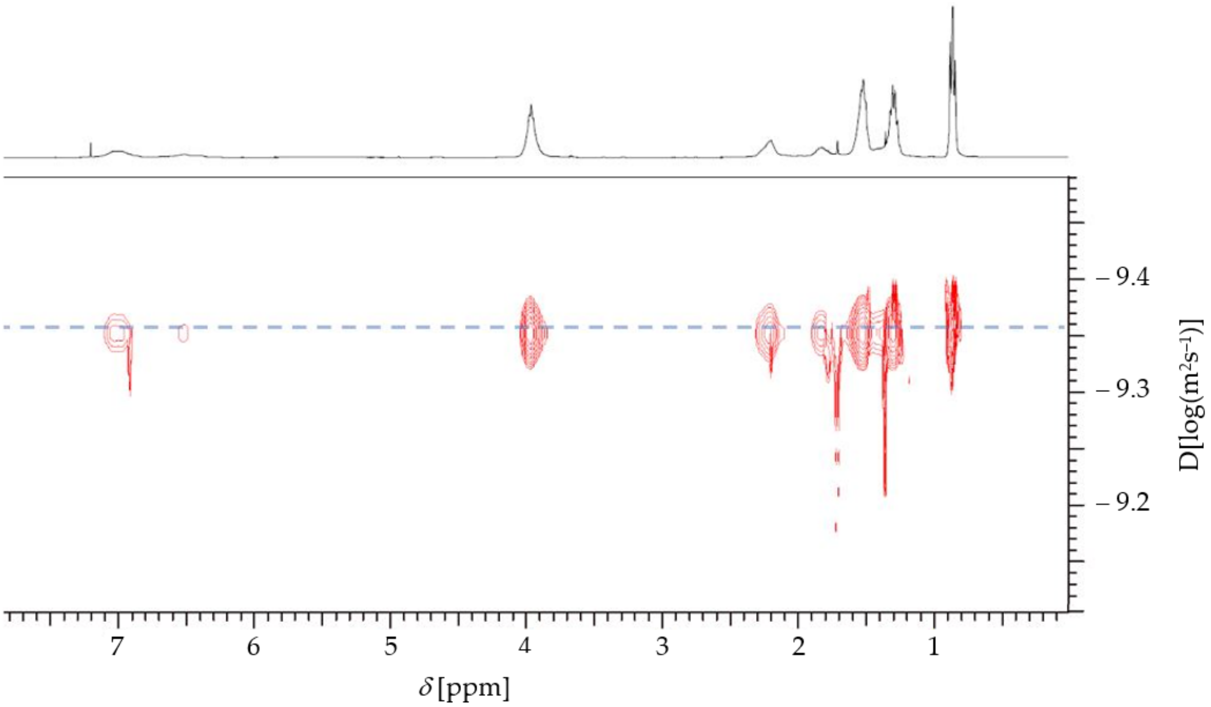

Figure S7: DOSY-NMR spectrum of PS-*b*-PBA-*b*-PS (**P2b**) in $CDCl_3$ (400 MHz).

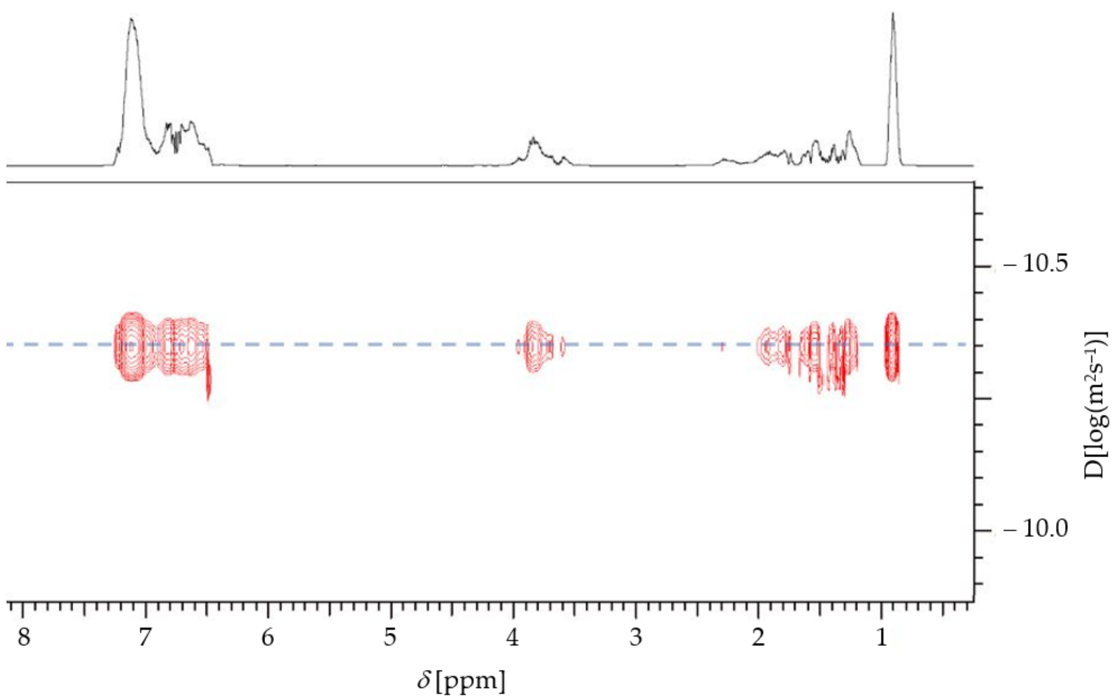

Figure S8: DOSY-NMR spectrum of PS-*b*-PBA-*b*-PS (**P3b**) in $CDCl_3$ (400 MHz).

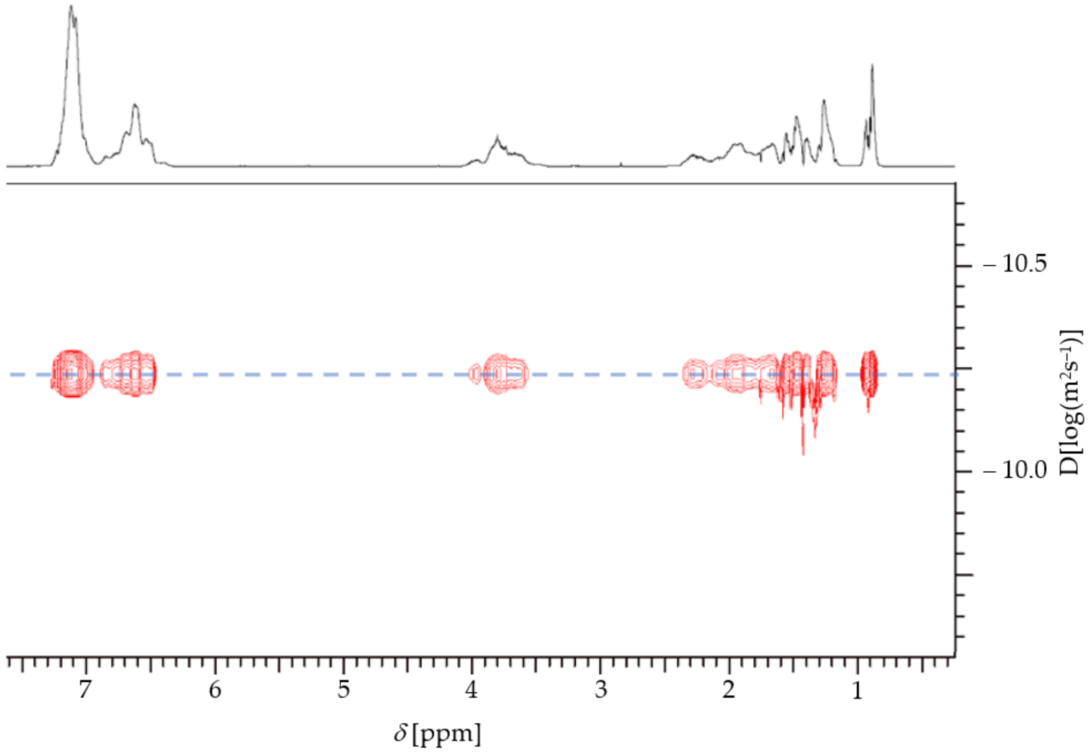

Figure S9. DOSY-NMR spectrum of PS-*b*-PBA-*b*-PS-*b*-PBA-*b*-PS (**P3c**) in $CDCl_3$ (500 MHz).

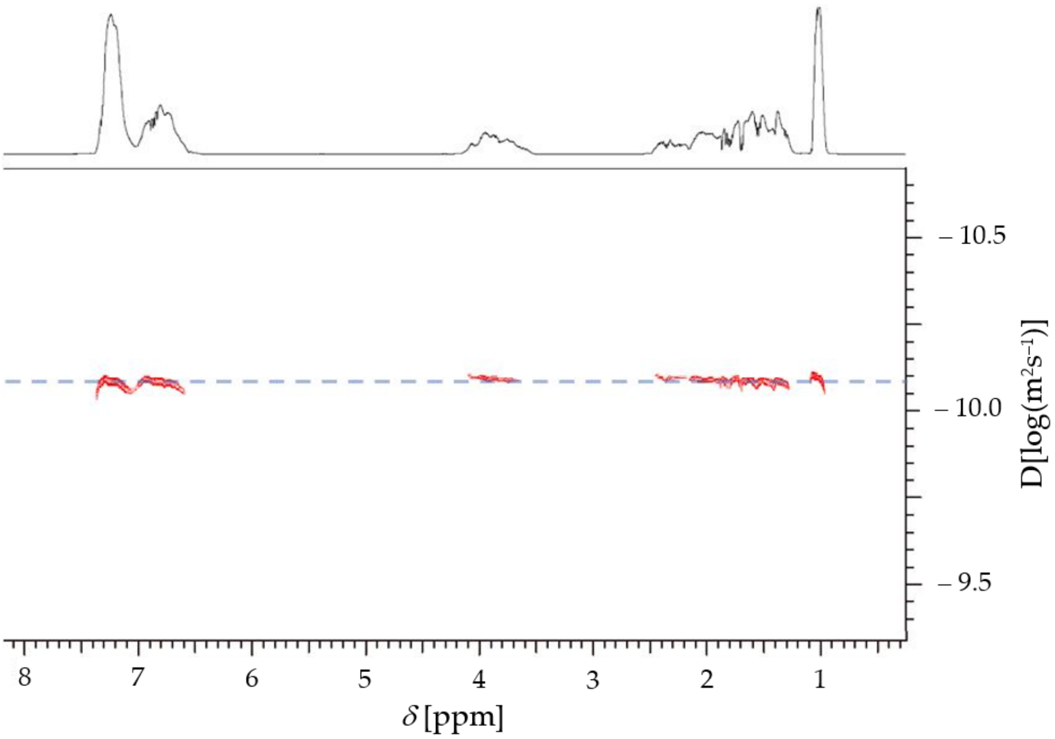

Figure S10. DOSY-NMR spectrum of PS-*b*-PBA-*b*-PS-*b*-PBA-*b*-PS (**P4c**) in $CDCl_3$ (500 MHz).

2.3. SEC-Diagrams

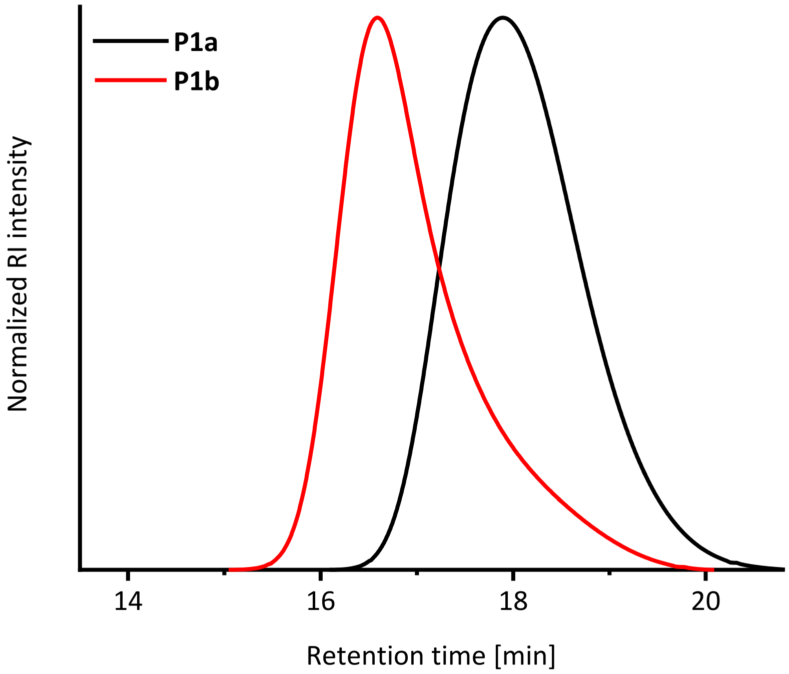

Figure S11: SEC-curves of the manually synthesized block copolymer **P1**. Black: First reaction step (PS, **P1a**). Red: Second reaction step (PS-*b*-PBA-*b*-PS, **P1b**), (chloroform/isopropanol/triethylamine [94/2/4], PS-standard).

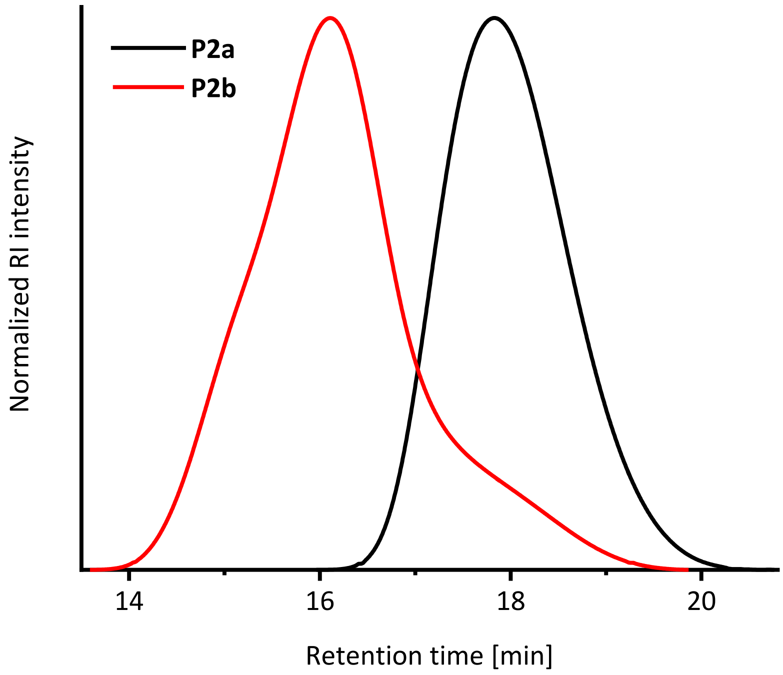

Figure S12: SEC-curve of the automatically synthesized block copolymer **P2**. Black: First reaction step (PS, **P2a**). Red: Second reaction step (PS-*b*-PBA-*b*-PS, **P2b**), (chloroform/isopropanol/triethylamine [94/2/4], PS-standard).

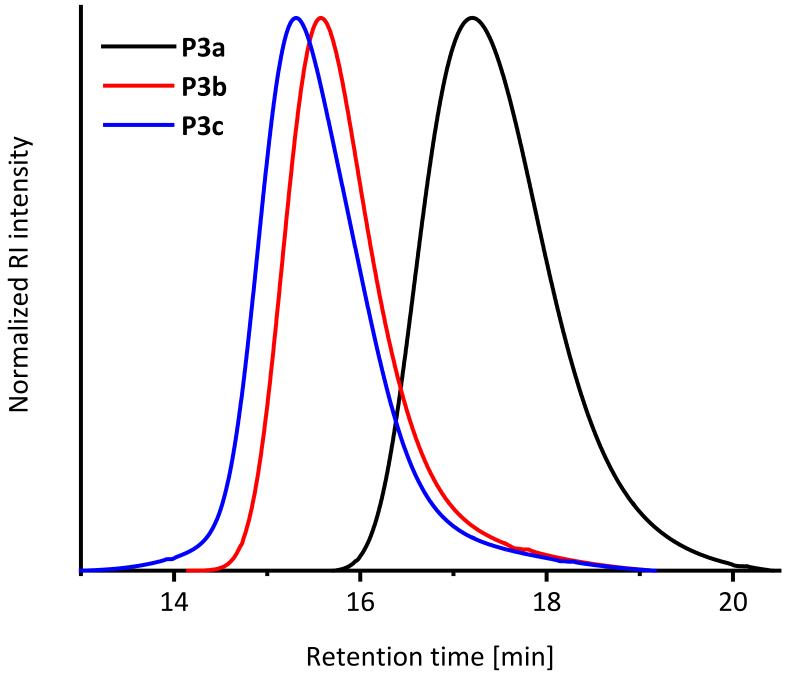

Figure S13: SEC-curve of the manually synthesized block copolymer **P3**. Black: First reaction step (PS, **P3a**). Red: Second reaction step (PS-*b*-PBA-*b*-PS, **P3b**). Blue: Third reaction step (PS-*b*-PBA-*b*-PS-*b*-PBA-*b*-PS, **P3c**), (chloroform/isopropanol/triethylamine [94/2/4], PS-standard).

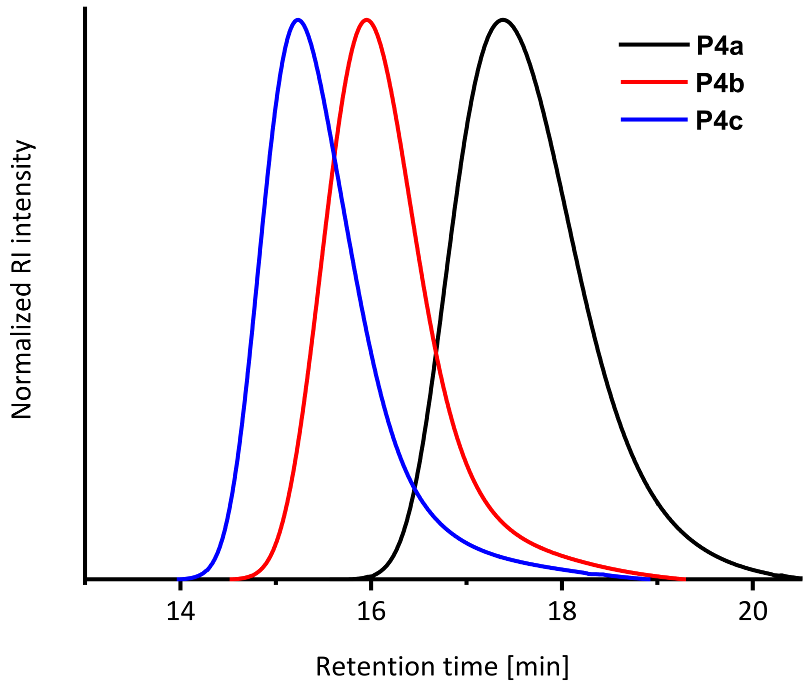

Figure S14: SEC-curve of the automatically synthesized block copolymer **P4**. Black: First reaction step (PS, **P4a**). Red: Second reaction step (PS-*b*-PBA-*b*-PS, **P4b**). Blue: Third reaction step (PS-*b*-PBA-*b*-PS-*b*-PBA-*b*-PS, **P4c**), (chloroform/isopropanol/triethylamine [94/2/4], PS-standard).

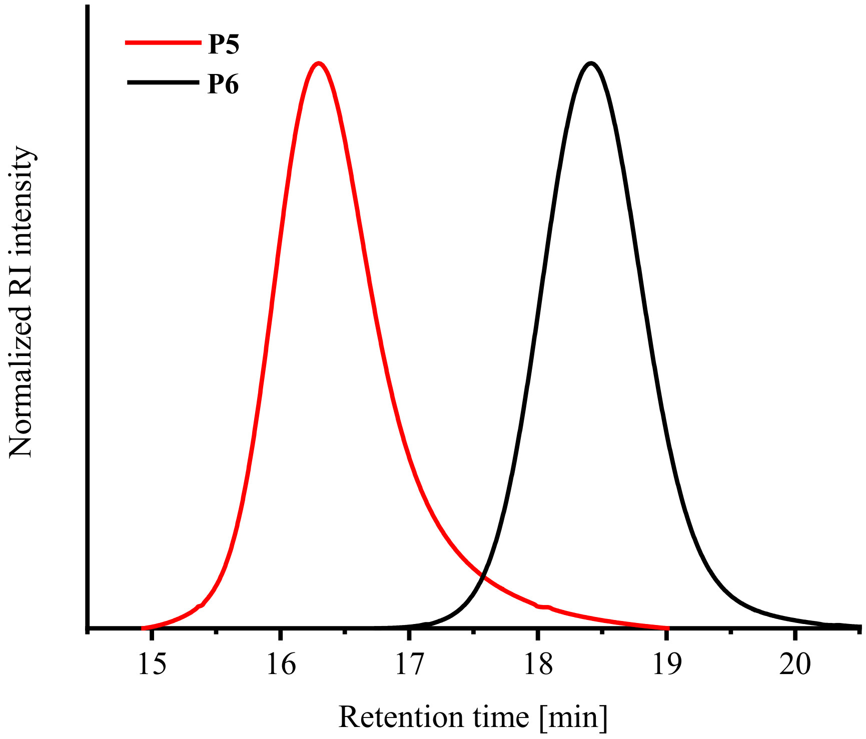

Figure S15: SEC-curves of the synthesized polymers **P5** and **P6**. Red: PMMA, **P5**. Black: PS, **P6**) (chloroform/isopropanol/triethylamine [94/2/4], PMMA or PS-standard).
